# Effects of different priming doses of propofol on fentanyl-induced cough during anesthesia induction: A preliminary randomized controlled study

**DOI:** 10.3109/03009730903291034

**Published:** 2010-04-07

**Authors:** Qifeng Tang, Yanning Qian, Qingwei Zhang, Jianjun Yang, Zhongyun Wang

**Affiliations:** ^1^Department of Anesthesiology, Affiliated Suzhou Hospital of Nanjing Medical University, SuzhouP. R. China; ^2^Department of Anesthesiology, First Affiliated Hospital of Nanjing Medical University, NanjingP. R. China; ^3^Department of Anesthesiology, Jinling Hospital, Nanjing University, NanjingP. R. China

**Keywords:** Anesthesia, cough, dose, fentanyl, propofol

## Abstract

Fentanyl-induced cough is not an uncommon condition during the induction of general anesthesia. A preliminary randomized controlled study was designed to observe the effects of different priming doses of propofol on fentanyl-induced cough during anesthesia induction. A total of 120 patients were randomized into 4 groups (*n* = 30) to receive the intravenous injection of intralipid (group I), propofol 1 mg·kg^-1^ (group II), propofol 1.5 mg·kg^-1^ (group III), or propofol 2 mg·kg^-1^ (group IV) 1 minute before a bolus of fentanyl 2.5 µg·kg^-1^. The occurrence and severity of cough were recorded for 2 minutes after fentanyl bolus. The severity of cough was graded as none (grade 0), mild (grade 1–2), moderate (grade 3–4), or severe (grade 5 or more). The average bolus time of fentanyl was 1.5 ± 0.3 seconds in the present study. The incidence of fentanyl-induced cough was 80.0% in group I, 40.0% in group II, 6.7% in group III, and 3.3% in group IV, respectively. Groups II, III, and IV had a lower incidence and less severity of cough than group I (*P* < 0.05). Groups III and IV had a lower incidence and less severity of cough than group II (*P* < 0.05). In summary, a priming dose of more than 1 mg·kg^-1^ of propofol is effective to suppress fentanyl-induced cough in a dose-dependent manner. We suggest using a priming dose of propofol 1.5 mg·kg^-1^ to suppress cough during the anesthesia induction with propofol and fentanyl in clinical practice.

## Introduction

Intravenous administration of fentanyl is widely used during the induction of general anesthesia. Fentanyl-induced cough is widely reported (1–4) but has not been considered as a serious anesthetic complication during anesthesia induction. Lin and colleagues (1) have shown a 65% incidence of cough in patients following intravenous administration of fentanyl 2.5 µg·kg^-1^ via a freely running peripheral venous catheter within 2 seconds. Böhrer and co-workers (2) have observed 45.9% of the patients cough after receiving fentanyl 7 µg·kg^-1^ through a central venous catheter, while only 2.7% of the patients cough after a bolus of fentanyl 7 µg·kg^-1^ via a peripheral cannula. Phua and co-authors (3) have demonstrated that the injection of fentanyl 1.5 µg·kg^-1^ via a peripheral venous line elicits cough in 28% of the patients, and a similar incidence of cough (33%) is documented by Shen and co-researchers (4) following a bolus of fentanyl 2 µg·kg^-1^ via a peripheral venous route within 5 seconds. The discrepancies in the incidence of cough among these studies may be attributed to the different doses, routes, and rates of fentanyl administration (1–4).

However, fentanyl-induced cough is not always benign and is undesirable in patients with some pre-existing diseases, including cerebral aneurysm, brain trauma, brain hernia, open eye injury, dissecting aortic aneurysm, pneumothorax, and hypersensitive airway disease (5). Various attempts have been made to reduce the incidence of fentanyl-induced cough during anesthesia induction (1,5–8). The primary effective drugs for suppressing this cough include lidocaine, ephedrine, β_2_-receptor agonist, ketamine, and clonidine (1,5–7). All these drugs have bronchorelaxant effects on airway smooth muscle (1,5–7). Propofol may also induce bronchodilation (9,10), therefore we hypothesized that an appropriate dose of propofol might suppress fentanyl-induced cough. So, we designed a preliminary randomized controlled study to observe the effects of different priming doses of propofol on fentanyl-induced cough during anesthesia induction.

## Materials and methods

### Patient population

The Ethics Committee of Affiliated Suzhou Hospital of Nanjing Medical University approved the protocol of the present study, and informed written consents were obtained from all patients.

A total of 120 patients of either sex, aged 25 to 60 years, American Society of Anesthesiologists (ASA) class I or II, scheduled for elective abdominal surgery with general anesthesia were enrolled in the present study. Exclusion criteria included: body-weight exceeding 20% of ideal body-weight (on the basis of body mass index recommended); impaired kidney or liver function; presence of a gastric tube; or a history of asthma, chronic cough, smoking, upper respiratory tract infection in the previous 2 weeks, or treated with angiotensin-converting enzyme inhibitors, bronchodilators, or steroids in the previous 2 weeks.

Dr Tang decided whether a patient should be included in the present study according to the inclusion and exclusion criteria, randomized the patients to 4 groups of 30 patients by using a computer-generated table of random numbers, and was responsible for drug preparation. The allocation sequences were contained in a set of sealed envelopes, and the observer and all the patients involved in the present study were blinded.

### Anesthesia induction and data collection

Phenobarbital sodium 0.1 g and atropine 0.5 mg were injected intramuscularly 30 min before anesthesia. In the operating room, venous access to the median cubital vein was established with an 18-gauge cannula. The vertical distance between the drip bottle and the venous access was 1 meter in all patients. Electrocardiogram, non-invasive blood pressure, and pulse oximeter were applied throughout the surgery. Patients were left undisturbed for more than 1 minute. Then, patients received intravenously anesthesia induction as the following sequence of medications: injecting intralipid (placebo) 0.15 mL·kg^-1^ in group I, propofol (10 mg·mL^-1^; AstraZeneca Co., Italy) 1 mg·kg^-1^ in group II, propofol 1.5 mg·kg^-1^ in group III, and propofol 2 mg·kg^-1^ in group IV; 1 minute later, bolusing fentanyl (50 μg·mL^-1^; Renfu Co., Hubei, China) 2.5 µg·kg^-1^ in all groups; 2 minutes after fentanyl bolus, pushing propofol 3, 2, 1.5, and 1 mg·kg^-1^ in groups I, II, III, and IV, respectively; and then injecting succinylcholine 2 mg·kg^-1^ in all groups. All the bolus time of fentanyl was controlled, less then 2 seconds, and was recorded in the present study. One-and-a-half minutes after anesthesia induction, all patients were successfully endotracheally intubated, and they underwent surgery favorably.

An observer, unaware of the type of medications given to the patients, recorded the occurrence and severity of cough for 2 minutes after fentanyl bolus. The severity of cough was graded as none (grade 0), mild (grade 1–2), moderate (grade 3–4), or severe (grade 5 or more).

### Statistical analysis

Data are expressed as mean ± SD, number, proportion, or percentage. Statistical analysis was performed by Statistical Product for Social Sciences (SPSS) software 13.0. The frequencies of cough and the proportions of sex and ASA class were compared using chi-square test or Fisher's exact test with Bonferroni correction. One-way analysis of variance was used to compare the age and weight among the four groups. *P* < 0.05 was considered statistically significant.

## Results

### Demographic characteristics

All patients completed the present study. There was no statistically significant difference between the four groups with regard to age, weight, sex, and ASA class (*P* > 0.05, Table I).

**Table I.** Demographics in four groups (*n* = 30).

Demographics	Group I	Group II	Group III	Group IV
Age, years	41 ± 6	42 ± 7	43 ± 6	42 ± 5
Weight, kg	54 ± 5	59 ± 8	58 ± 6	57 ± 7
Sex (male/female), *n*	12/1	11/19	10/20	12/18
ASA class (I/II), *n*	16/14	18/12	15/15	19/11

Data are expressed as mean ± SD or number (proportion). There was no significant difference with regard to demographics between the four groups. ASA = American Society of Anesthesiologists.

### Incidence and severity of fentanyl-induced cough

The average bolus time of fentanyl was 1.5 ± 0.3 seconds in the present study. The incidence of fentanyl-induced cough was 80.0% in group I, 40.0% in group II, 6.7% in group III, and 3.3% in group IV. Groups II, III, and IV had a lower incidence and less severity of cough than group I (*P* < 0.05). Groups III and IV had a lower incidence and less severity of cough than group II (*P* < 0.05). There was no significant difference in the incidence and severity of cough between groups III and IV (*P* > 0.05) (Table II).

**Table II.** Fentanyl-induced cough and its severity in four groups (*n* = 30) given intralipid or different priming doses of propofol.

Cough	Group I	Group II ^a^	Group III ^a,b^	Group IV ^a,b^
Incidence	24 (80.0)	12 (40.0)	2 (6.7)	1 (3.3)
Severity				
None	6 (20.0)	18 (60.0)	28 (93.3)	29 (96.7)
Mild	15 (50.0)	6 (23.3)	2 (6.7)	1 (3.3)
Moderate	7 (23.3)	4 (13.3)	0 (0)	0 (0)
Severe	2 (6.7)	2 (3.3)	0 (0)	0 (0)

Data are expressed as number (percentage). ^a^ *P* < 0.05, groups II, III, and IV versus group I. ^b^ *P* < 0.05, groups III and IV versus group II.

## Discussion

The major finding in the present study was that the pretreatment with propofol 1, 1.5, and 2 mg·kg^-1^ might reduce fentanyl-induced cough from 80.0% to 40.0%, 6.7%, and 3.3%, respectively, which was consistent with our hypothesis.

Various underlying mechanisms of fentanyl-induced cough have been proposed without definite conclusion. Pulmonary chemoreflexes mediated by either vagal C-fiber receptors in close proximity to pulmonary vessels (11,12) or by irritant receptors have been suggested (2,12). For example, fentanyl may cause vagal predominance and induce reflex bronchoconstriction and cough (10–13). Effectively reducing fentanyl-induced cough response from 28% to 6% after aerosol inhalation of salbutamol (a selective β_2_-adrenergic bronchodilator) further supports the mechanism that fentanyl induces cough via its bronchoconstriction effect (5). In addition, opioid-induced histamine release (14) and muscle rigidity leading to sudden adduction of the vocal cords or supraglottic obstruction (15) are plausible explanations.

Propofol possesses bronchodilation effect (9,10,16,17). Burburan and colleagues (16) have concluded that propofol inhibits bronchoconstriction and decreases the risk of bronchospasm during anesthesia induction. Pizov and co-workers (17) have shown that the incidence of wheezing was significantly reduced in asthmatic patients receiving a propofol-based induction of anesthesia compared to a barbiturate-based induction. Additionally, propofol has a significant sedative effect that may also reduce the incidence of cough (7). Therefore, propofol may be a promising drug to suppress fentanyl-induced cough. In the present study, we observed that a priming dose of 1 mg·kg^-1^ of propofol was able to suppress fentanyl-induced cough significantly and higher doses of propofol (1.5–2 mg·kg^-1^) were more effective in suppressing fentanyl-induced cough. However, Lin and colleagues (1) observed that premedication with propofol 0.6 mg·kg^-1^ could not inhibit fentanyl-induced cough significantly. So, we presume that propofol may suppress fentanyl-induced cough in a dose-dependent manner.

The incidence of fentanyl-induced cough varies over a wide range of 2.7%–65% and primarily depends on the doses of fentanyl injected, the rates of injection, and the routes of injection (1–4). In the present study, the incidence of cough was 80%. The exact reasons for this higher incidence of cough remained unclear. We attribute this higher incidence to the faster bolus speed of fentanyl (bolus time: 1.5 ± 0.3 seconds) and possible bias due to the small sample size included in the present study. So, fentanyl should not be injected with such rapidity in clinical practice.

It is interesting that the incidence of cough after fentanyl administration appears to be higher in Asians compared to Europeans (1–7,18). For example, Schäpermeier and Hopf (18) have observed that the incidence of cough following intravenous fentanyl 1.5 µg·kg^-1^ over 2, 5, or 10 seconds is between 3% and 6% in European patients; whereas Phua and co-authors (3) have demonstrated that injection of the same dose of fentanyl (1.5 µg·kg-^1^) elicits cough in 28% of the Asian patients.

There are two major limitations in interpreting the results of the present study. First, the total dose of 3 mg·kg^-1^ of propofol may be somewhat mildly overdosed even if the priming doses are commonly clinically used, and the time interval between priming and supplementing dose of propofol is relatively long (more than 3 minutes). Second, even if all groups are given the same premedication with phenobarbital sodium 0.1 g and atropine 0.5 mg, dosing per kg body-weight may be unequally distributed between groups, thus dosing of these drugs may be scientific confounders.

In conclusion, a priming dose of more than 1 mg·kg^-1^ of propofol given 1 minute before fentanyl is effective to suppress fentanyl-induced cough in a dose-dependent manner. Higher doses of propofol (1.5–2 mg·kg^-1^) are more effective in suppressing fentanyl-induced cough. Considering that higher doses of propofol are associated with a higher incidence of cardiovascular events, we suggest using a priming dose of propofol 1.5 mg·kg^-1^ to suppress cough during the anesthesia induction with propofol and fentanyl in clinical practice.

## References

[1] Lin CS, Sun WZ, Chan WH, Lin CJ, Yeh HM, Mok MS (2004). Intravenous lidocaine and ephedrine, but not propofol, suppress fentanyl-induced cough. Can J Anaesth.

[2] Böhrer H, Fleischer F, Werning P (1990). Tussive effect of a fentanyl bolus administered through a central venous catheter. Anaesthesia.

[3] Phua WT, Teh BT, Jong W, Lee TL, Tweed WA (1991). Tussive effect of a fentanyl bolus. Can J Anaesth.

[4] Shen JC, Xu JG, Zhou ZQ, Liu HJ, Yang JJ (2008). Effect of equivalent doses of fentanyl, sufentanil or remifentanil on incidence and severity of cough in 315 patients: a randomized, double-blind study. Curr Ther Res Clin E.

[5] Agarwal A, Azim A, Ambesh S, Bose N, Dhiraj S, Sahu D (2003). Salbutamol, beclomethasone or sodium chromoglycate suppress coughing induced by iv fentanyl. Can JAnaesth.

[6] Yeh CC, Wu CT, Huh BK, Lee MS, Lin SL, J Sheen M (2007). Premedication with intravenous low-dose ketamine suppresses fentanyl-induced cough. J Clin Anesth.

[7] Horng HC, Wong CS, Hsiao KN, Huh BK, Kuo CP, Cherng CH (2007). Pre-medication with intravenous clonidine suppresses fentanyl-induced cough. Acta Anaesthesiol Scand.

[8] Lin JA, Yeh CC, Lee MS, Wu CT, Lin SL, Wong CS (2005). Prolonged injection time and light smoking decrease the incidence of fentanyl-induced cough. Anesth Analg.

[9] Cheng EY, Mazzeo AJ, Bosnjak ZJ, Coon RL, Kampine JP (1996). Direct relaxant effects of intravenous anesthetics on airway smooth muscle. Anesth Analg.

[10] Yamaguchi M, Shibata O, Nishioka K, Makita T, Sumikawa K (2006). Propofol attenuates ovalbumin-induced smooth muscle contraction of the sensitized rat trachea: inhibition of serotonergic and cholinergic signaling. Anesth Analg.

[11] Yasuda I, Hirano T, Yusa T, Satoh M (1978). Tracheal constriction by morphine and by fentanyl in man. Anesthesiology.

[12] Fukuda K, Miller RD (2005). Intravenous opioid anesthetics. Miller's anesthesia.

[13] Stellato C, Cirillo R, de Paulis A, Casolaro V, Patella V, Mastronardi P (1992). Human basophil/mast cell releasability. IX. Heterogeneity of the effects of opioids on mediator release. Anesthesiology.

[14] Lin JA, Chen FC, Lee MS, Horng HC, Cherng CH, Yeh CC (2007). Intravenous dexamethasone pretreatment reduces fentanyl-induced cough. J Formos Med Assoc.

[15] Benthuysen JL, Smith NT, Sanford TJ, Head N, Dec-Silver H (1986). Physiology of alfentanil-induced rigidity. Anesthesiology.

[16] Burburan SM, Xisto DG, Rocco PR (2007). Anaesthetic management in asthma. Minerva Anestesiol.

[17] Pizov R, Brown RH, Weiss YS, Baranov D, Hennes H, Baker S (1995). Wheezing during induction of general anesthesia in patients with and without asthma. A randomized, blinded trial. Anesthesiology.

[18] Schäpermeier U, Hopf HB (2008). Fentanyl-induced cough does not depend on injection speed: a randomized study. Acta Anaesthesiol Scand.

